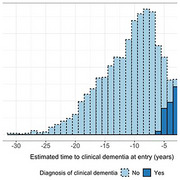# Prevalence of dementia within economic burden of east‐African region of Uganda

**DOI:** 10.1002/alz70858_096873

**Published:** 2025-12-24

**Authors:** Waiswa Moses, Steven Brunswerk, Henry Kakooza, Jackline Nakalembe, Denis Ssekikubo

**Affiliations:** ^1^ Iganga General Hospital, Kampala, Uganda; ^2^ Makerere University department for health policy, Kampala, Uganda; ^3^ National Center for health statistics, Kampala, Uganda; ^4^ National population policy for social transformation, Kampala, Uganda; ^5^ Ministry of Finance and Population health, Kampala, Uganda

## Abstract

**Background:**

The growing prevalence of dementia is a global concern, especially in the East‐African Uganda, Kenya, Tanzania, where updated economic impact data are scarce. Understanding its prevalence and cost is crucial for effective polices and support systems.

**Method:**

United Nations population data and dementia prevalence estimates were used to calculate total cases. Direct costs were based on gross domestic product (GDP) per capita (purchasing power parity) and income classification. Indirect caregiver support costs were estimated using average monthly wages and two distinct scenarios.

**Result:**

The highest dementia prevalence among those aged more than 60 years was in Uganda (4.88%), Tanzania (4.43%) and Kenya (4.19%). The total direct cost in the East African region was $8.18 billion for those over 50 years old. Indirect costs ranged from $2.25 billion (best case) to $5.67 billion (worst case), with a mean value of $3.98 billion. Total dementia care costs (direct and indirect) under the mean scenario for the entire east African region amounted to $12.17 billion, with costs as a percentage of GDP ranging from 0.05% (Uganda) to 0.44% (Kenya

**Conclusion:**

This study highlights dementia as a growing public health issue in the East African region, with 1 329 729 individuals affected in 2021 and total costs between $10.43 billion and $13.90 billion. The findings emphasize the urgent need for investment in research and specialised services for older adults, particularly those with dementia. With the projected increase in the prevalence of dementia in the coming decades, addressing this public health challenge is critical to ensuring the well‐being of older adults and reducing the economic burden on societies in the region.